# Pharmacokinetics and Safety of Group A and B Anti-Tuberculosis Drugs Used in Treatment of Rifampicin-Resistant Tuberculosis during Pregnancy and Post-Partum: A Narrative Review

**DOI:** 10.3390/pathogens12121385

**Published:** 2023-11-24

**Authors:** Jennifer Hughes

**Affiliations:** Desmond Tutu TB Centre, Department of Paediatrics and Child Health, Faculty of Medicine and Health Sciences, Stellenbosch University, Cape Town 7500, South Africa; jhughes@sun.ac.za

**Keywords:** pharmacokinetics, safety, rifampicin-resistant tuberculosis, pregnancy, post-partum

## Abstract

Recommendations for treatment of rifampicin-resistant tuberculosis (RR-TB) during pregnancy and post-partum now include Group A and B antituberculosis drugs. While pharmacokinetic data for most of these drugs among adults receiving treatment for RR-TB are limited, the data from pregnant patients and their infants are extremely scarce. Existing data suggest that fluoroquinolones, bedaquiline, clofazimine and terizidone may be used safely in pregnancy. Pharmacokinetic exposures, particularly between trimesters, are potentially sub-optimal; however, there is currently no evidence to support dose adjustment during pregnancy. Linezolid poses a potentially serious toxicity risk, particularly as exposures appear to be high in the later stages of pregnancy and post-partum following extended use, but this should be considered alongside the benefits of this extremely effective drug in the treatment of this life-threatening disease. While plenty of questions remain regarding the exposure to Group A and B antituberculosis drugs through breastmilk, existing literature suggests minimal harm to the breastfed infant. Pregnant patients and their infants should be included in therapeutic trials and pharmacokinetic studies of effective antituberculosis drugs.

## 1. Introduction

Untreated tuberculosis is associated with high mortality, particularly during pregnancy [1]. Rifampicin-resistant tuberculosis (RR-TB), which includes multidrug-resistant (MDR)-TB, a disease caused by a strain of *Mycobacterium tuberculosis* resistant to rifampicin and isoniazid with or without resistance to other antituberculosis drugs, is detected in almost 4% of all new tuberculosis cases and an estimated 20% of previously treated cases per year worldwide [2]. A considerable proportion of the estimated 450,000 incident cases of MDR/RR-TB in 2021 would have occurred among people of child-bearing potential, some of whom were likely to be pregnant around the time that they would have required treatment for the disease. Early diagnosis and the initiation of an efficacious multidrug treatment, along with the prompt identification and appropriate management of adverse drug effects, is crucial to improve maternal tuberculosis treatment outcomes and prevent the transmission of tuberculosis to the foetus/infant [3].

Recommendations for the treatment of MDR/RR-TB in adults have changed dramatically over the past decade, with the introduction of novel and repurposed agents, the advent of injectable-free, standardised treatment regimens, and a shorter duration of treatment from 24 to 6 months [4]. Until recently, the general guidance for the treatment of MDR/RR-TB during pregnancy has been to avoid novel and repurposed agents due to the lack of safety and efficacy data in this population, and to use drugs with a better-established safety profile [5]. While potential harm to the foetus is a key consideration during MDR/RR-TB treatment, a tolerable regimen composed of efficacious drugs is crucial for the successful cure of the disease and survival of the child’s birth-parent [6]. The latest World Health Organization (WHO) guidelines [4] recommend a six-month regimen consisting of bedaquiline–pretomanid–linezolid, with or without a fluoroquinolone, for the treatment of MDR/RR-TB in people aged ≥15 years, but not during pregnancy. The standardised 9-month all-oral regimen, consisting of bedaquiline–linezolid–levofloxacin (or moxifloxacin)–clofazimine–ethambutol–pyrazinamide–high-dose isoniazid, may be used during pregnancy and post-partum for patients who meet specific eligibility criteria [4].

The primary concern regarding the use of second-line antituberculosis drugs during pregnancy and post-partum, over and above the toxicity risks known to be associated with long-term use in all patients receiving treatment for MDR/RR-TB, is usually for the safety of the foetus/infant exposed to these drugs in utero or during breastfeeding. While there are several methods of assessing maternal–foetal drug transfer in utero, such invasive procedures are not performed routinely during most pregnancies, and the collection of drug concentration data is limited by difficulties associated with obtaining specimens from relevant anatomical sites [7]; therefore, published data on foetal exposure to anti-tuberculosis drugs in utero are rare. After birth, infants can be assessed clinically for adverse events possibly related to exposure to specific drugs, in utero and through breastfeeding, and the co-ordinated pharmacokinetic sampling of foetal blood, umbilical cord blood, maternal blood and/or breastmilk can provide estimates of the extent of foetal exposure to such drugs [7]. A recent systematic literature review found that published data on maternal plasma and breastmilk concentrations and infant exposures to most second-line antituberculosis drugs are extremely scarce [8].

Another important concern is the relative lack of information from the pregnant and post-partum period to inform the optimal dosing of second-line antituberculosis drugs required to achieve efficacious maternal drug exposures, which may be influenced by physiological changes during and after pregnancy. The effects of pregnancy on the pharmacokinetics of drugs include the following: increased plasma volume and body fat and changes in drug-binding plasma protein concentrations, which affect the volume of distribution of many drugs; metabolism is altered by the increased or decreased activity of drug-metabolising enzymes and transporter proteins; greater cardiac output and blood flow to organs can result in increased hepatic clearance or renal elimination through increased glomerular filtration and creatinine clearance [9]. The effects of the gastrointestinal changes of pregnancy on drug absorption appear to be relatively limited [9]. These pharmacokinetic variations due to physiological changes during pregnancy can affect plasma concentrations (as measured via the maximum and minimum plasma concentration (C_max_ and C_min_, respectively) of a drug between dosing intervals) and overall drug exposures (as measured by the area under the concentration–time curve (AUC) for a select period between dosing intervals) [10].

In people requiring treatment for MDR/RR-TB disease, the main risk associated with sub-optimal plasma concentrations and low drug exposures of antituberculosis drugs is that the antimycobacterial activity and intended therapeutic efficacy of the drug may be compromised and thereby lower the chance of a successful treatment outcome [11]. The other concern is that sub-optimal drug concentrations and exposures may drive further acquisition of drug resistance of the *M. tuberculosis* isolate through selective drug pressure with an otherwise inadequate or compromised treatment regimen [12]. Conversely, increased plasma concentrations and high drug exposures may result in increased toxicity, reduced tolerability and subsequent poor adherence to the antituberculosis therapy. The potential risks associated with sub-optimal or excessive drug exposures are amplified in pregnancy as the potential maternal and foetal consequences are inextricably linked.

Pregnancy tends to be an exclusion criterion in most studies evaluating novel drugs and shorter, safer and more efficacious treatment regimens for people with MDR/RR-TB; as a result, specific dosing and safety data are severely lacking in this vulnerable population who still require treatment for the disease. A review of the pharmacokinetics and safety (for mother and foetus/infant) of WHO Group A and B antituberculosis drugs in the treatment of MDR/RR-TB during pregnancy and post-partum is presented here.

## 2. WHO Categorization of Anti-Tuberculosis Medications

In 2018, the WHO categorised medications for treatment of MDR/RR-TB into three groups based on efficacy data among adults (Table 1) [5]. Drugs from WHO Group A (levofloxacin/moxifloxacin, bedaquiline and linezolid) are independently associated with better treatment outcomes and improved survival and are therefore prioritised in all regimens where possible. Drugs from Group B (clofazimine and cycloserine/terizidone) are associated with more favourable treatment outcomes but no significant effect on survival. Other potentially effective antituberculosis medications are included in Group C (or remain uncategorised) due to limited data on their independent effect on MDR/RR-TB treatment outcomes. Patients (including those pregnant and post-partum) who are not eligible to receive the shorter (6–9 months), standardised regimens for MDR/RR-TB are usually treated with individualised regimens for longer periods (12–18 months). The design of such regimens involves the selection of at least four drugs from WHO Groups A and B, with consideration of the likely efficacy, safety and tolerability of specific drugs for the individual patient; Group C drugs are considered only if options are otherwise limited.

Common side effects of antituberculosis drugs and potential concerns and considerations during pregnancy and post-partum are summarised in Table 1.

## 3. WHO Group A Drugs

### 3.1. Fluoroquinolones (Levofloxacin and Moxifloxacin)

Fluoroquinolones work through inhibiting bacterial DNA replication, transcription, recombination and repair [19]. Their bactericidal activity is concentration-dependent [20] and, therefore, C_max_ and AUC (usually over 24 h: AUC_0–24_) are important pharmacokinetic parameters for this class of drugs. Peloquin et al. [21] previously described the pharmacokinetic parameters among non-pregnant adults with confirmed tuberculosis receiving the upper range of recommended daily doses of levofloxacin (1000 mg, n = 10) or moxifloxacin (400 mg, n = 9) as monotherapy over seven days. Both drugs were rapidly absorbed within the first hour, with C_max_ in the range of 9 to 43 mcg/mL, AUC_0–24_ (free drug, unbound to plasma proteins) in the range of 77.26 to 295.86 mg·h^−1^·L^−1^ and a median elimination half-life (t_½_) of 7.37 h for levofloxacin and a C_max_ range of 4.47 to 9.00 mcg/mL, AUC_0–24_ range of 18.32 to 39.13 mg·h^−1^·L^−1^ and t_½_ of 6.66 h for moxifloxacin [21]. Levofloxacin is cleared primarily via renal elimination, whereas moxifloxacin is cleared hepatically and renally [11]; therefore, the clearance of both drugs may be increased due to relevant physiological changes in pregnancy [10]. Compared to non-pregnant individuals, circulating concentrations of fluoroquinolones appear to be generally reduced in pregnant women exposed to these drugs for conditions other than tuberculosis [19,22].

As reported by Shiu et al. in a qualitative review of the clinical pharmacokinetics and pharmacodynamics of anti-tubercular drugs in pregnancy in 2021 [23], the only published data on the pharmacokinetics of fluoroquinolones specifically in the context of tuberculosis treatment during pregnancy or post-partum (as remains the case to date) come from an observational case study of a single patient exposed to moxifloxacin (along with linezolid, pyrazinamide and prothionamide) through the second and third trimesters of pregnancy and post-partum [24]. Pharmacokinetic sampling was carried out (at steady state, >14 days into treatment) after 25 + 5 weeks and 35 + 5 weeks of gestation, and again after 18 weeks post-partum. At the standard WHO-recommended daily dose of 400 mg, moxifloxacin exposure over 24 h (AUC_0–24_) was reported as 31.6 mg·h^−1^·L^−1^ in the second trimester, 32 mg·h^−1^·L^−1^ in the third trimester and 34.9 mg·h^−1^·L^−1^ 18 weeks post-partum [24]. The authors suggested that the slightly lower moxifloxacin exposures during pregnancy could possibly be attributed to the increased volume of distribution and higher rate of elimination of the drug. However, the moxifloxacin exposures at all three stages pre- and post-partum were within the range of exposures measured in an earlier cohort of 16 non-pregnant adults from the same setting treated with the same daily dose of moxifloxacin for tuberculosis (median AUC_0–24_ was 24.8 mg·h^−1^·L^−1^, interquartile range [IQR] 20.7–35.2) [25].

Fluoroquinolones are highly protein-bound, but only the unbound drug has an antimicrobial effect, which is an important consideration in pregnancy, where levels of drug-binding plasma proteins (albumin and alpha-1-acid glycoprotein) are considerably reduced [26]. Furthermore, several studies have reported considerable variation in the protein binding of moxifloxacin [25] and levofloxacin [27] among non-pregnant adults treated for tuberculosis. Low fluoroquinolone exposures have been associated with the development of fluoroquinolone-resistant strains of *M. tuberculosis* [12], and there is concern that the currently recommended daily doses of fluoroquinolones (750–1000 mg for levofloxacin and 400 mg for moxifloxacin) for the treatment of MDR/RR-TB may be sub-optimal [28]. Population pharmacokinetic modelling data from 178 adults receiving daily doses of levofloxacin (500–1000 mg) or moxifloxacin (400–800 mg) for the treatment of MDR-TB indicated that much higher doses of levofloxacin (>1500 mg) and moxifloxacin (≥800 mg) may be required to achieve the maximum kill rate of mycobacteria at the minimum inhibitory concentration (MIC) of 1.0 mg/L and 0.25 mg/L, respectively [29], above which mycobacterial isolates have phenotypically detectable acquired resistance mechanisms. Increasing levofloxacin doses from 11 mg/kg up to 20 mg/kg results in proportionally increased exposures (median AUC_0–24_ [range] from 109 mcg·h^−1^·mL^−1^ [69–204] to 207 mcg·h^−1^·mL^−1^ [143–534]) [30]; however, due to the considerable pharmacokinetic variability of fluoroquinolones, individuals who are at risk of inadequate drug exposures, such as in pregnancy, may benefit from drug-concentration monitoring during treatment for MDR/RR-TB.

Fluoroquinolones are known to cross the placenta and enter the foetal compartment. Özyüncü et al. [31] measured maternal blood and amniotic fluid levels of levofloxacin and moxifloxacin two hours after a single, oral, prophylactic dose of each drug (levofloxacin 500 mg, n = 10; moxifloxacin 400 mg, n = 10) in women undergoing diagnostic amniocentesis in their second trimester. The reported mean (±standard deviation (SD)) maternal plasma concentrations of levofloxacin and moxifloxacin were 3.95 ± 0.77 mcg/mL and 3.53 ± 0.65 mcg/mL, respectively, with low rates of fluoroquinolone passage from maternal plasma to amniotic fluid (16% and 8%, respectively) [31]. The same group also measured fluoroquinolone concentrations in maternal and placental blood samples taken during Caesarean section, 30 min after the intravenous infusion of levofloxacin (dose 500 mg, n = 12) or moxifloxacin (dose 400 mg; n = 10). Mean (±SD) maternal plasma concentrations were 8.18 ± 1.68 mcg/mL for levofloxacin and 4.96 ± 1.36 mcg/mL for moxifloxacin, with high mean transplacental transfer rates of 67% and 75%, respectively, from the maternal plasma to the foetal venous circulation. However, the high transfoetal passage rates of levofloxacin (84%) and moxifloxacin (91%), measured by comparing concentrations in the foetal venous and arterial circulatory systems, indicate that foetal drug exposure in utero is low, as <15% of the drug remains in the foetus [32].

Fluoroquinolones are often avoided during pregnancy due to conflicting evidence of teratogenicity in animals and concerns over foetal cartilage development [33], but several systematic reviews have reported no association between quinolones and foetal malformations or other adverse pregnancy outcomes [13]. A subsequent observational study reported that levofloxacin was a significant predictor of low birth weight among 108 pregnant women treated for MDR/RR-TB [15].

Fluoroquinolones are also excreted in human breastmilk [19]. In a case report of a woman treated for infection (not tuberculosis) immediately post-partum with the intravenous followed by oral administration of 500 mg levofloxacin daily for 23 days, pharmacokinetic modelling estimates indicated a peak levofloxacin concentration of 8.2 mcg/mL in breast milk five hours post dose, t_1/2_ of seven hours and AUC_0–24_ of 120 mg·h/L [34]. No maternal blood was taken to calculate the milk–plasma concentration ratio; however, an earlier study of older-generation quinolone exposure in pregnant and lactating women indicated high concentrations in breastmilk (>75% relative to maternal plasma concentrations even two hours after dosing) [14]. The clinical implications of fluoroquinolone exposure in breastmilk have not been widely reported; however, based on available data, the risks to the breastfeeding infant are considered to be negligible given the extremely low estimated dose delivered to the infant through breastmilk in this case [8].

### 3.2. Bedaquiline

The pharmacokinetics and safety profile of this diarylquinolone, which works through inhibiting mycobacterial ATP-synthase, have been well described in non-pregnant adults and children with MDR/RR-TB [35]. The drug is absorbed relatively slowly over 5–6 h following oral administration, but absorption is enhanced two-fold with concurrent food intake. It is also highly protein-bound in plasma, which may have implications for concentrations achieved during pregnancy due to changes in plasma protein concentrations [10]. Among non-pregnant adults receiving treatment for MDR/RR-TB, the reported mean (±SD) C_max_ is 2.763 ± 1.185 mg/L after two weeks of oral daily dosing at 400 mg, and 1.267 ± 0.435 mg/L after a subsequent 22 weeks of bedaquiline dosed at 200 mg thrice weekly, with a mean target plasma concentration of 600 ng/mL at a steady state [36]. The drug is widely distributed in body tissues, resulting in a long terminal half-life exceeding five months. Bedaquiline is metabolised in the liver and is affected by changes in the activity of the cytochrome P-450 isoenzyme 3A4 (CYP3A4) system [37], which has implications for the metabolism of bedaquiline during pregnancy and post-partum. The active metabolite M2 has a much weaker therapeutic effect against *M. tuberculosis* but still contributes to the toxicity profile of the drug. As bedaquiline and the M2 metabolite are mostly excreted in stool, elimination is unlikely to be affected by ante- and post-partum changes in renal function.

Since a 2021 review of pharmacokinetics and pharmacodynamics of antituberculosis drugs in pregnancy [23], Court et al. [16] published the first human data on bedaquiline exposures among 13 pregnant women with MDR/RR-TB, as well as post-partum pharmacokinetic parameters in six women and four infants. Although the median C_max_ of 1.69 mg/L (range 0.296–2.93) recorded among five women sampled at six hours post bedaquiline dose in the third trimester of pregnancy is comparable to C_max_ reported in non-pregnant adults with MDR/RR-TB, overall ante-partum bedaquiline exposures were lower than expected [16]. Bedaquiline appears to be the only drug from WHO Groups A or B for which pharmacokinetic parameters have been modelled during MDR/RR-TB treatment in pregnancy and post-partum. The data presented did not fit the existing model well, despite the re-estimation of multiple parameter values; this clearly highlights the need for more data to properly describe the pharmacokinetic parameters of bedaquiline and other antituberculosis drugs in this population. Six women and four infants had post-partum pharmacokinetic sampling (very similar maternal plasma concentrations were reported pre- and post-partum), but only one breastfeeding infant had corresponding maternal blood and breastmilk concentration data available. While bedaquiline concentrations in non-breastfed infants were lower than maternal plasma concentrations, the infant and maternal plasma bedaquiline concentrations were similar in the one breastfed infant, indicating considerable exposure to bedaquiline in breastmilk [16]. This poses an unproven but theoretical risk of monotherapy in infants infected and diagnosed with MDR/RR-TB in the post-partum period, in addition to the potential risk of bedaquiline-related adverse events (Table 1) among breastfeeding infants. There are no published data on adverse events experienced by infants or children exposed to bedaquiline under five years of age. In an observational study of 108 pregnant women treated for MDR/RR-TB, bedaquiline exposure in utero was an independent predictor of low birth weight; however, babies exposed to bedaquiline in utero were more likely to be thriving with normal development after 12 months compared to those who were not exposed [15].

### 3.3. Linezolid

Despite the inclusion of linezolid (an oxazolidinone that inhibits bacterial peptide synthesis) in virtually all recommended regimens for the treatment of anyone with MDR/RR-TB, published pharmacokinetic data for linezolid (for any indication) during pregnancy are severely lacking. Heidari et al. noted that linezolid plasma concentrations are usually higher in females than males due to lower drug clearance, volume of distribution and body weight [38]. However, these parameters are also affected by physiological changes in pregnancy (e.g., increased plasma volume and glomerular filtration rate [10]) and so the effects of linezolid in pregnant individuals remain unclear. Still, to date, the only published pharmacokinetic data for linezolid as part of MDR/RR-TB treatment during pregnancy comes from a case study report described earlier [24]. Linezolid was dosed orally at 300 mg twice daily (the current WHO recommendation for adults is 600 mg once daily) and a large variation in linezolid exposures was observed between the second-trimester, third-trimester and post-partum pharmacokinetic sampling timepoints. The linezolid exposures (AUC_0–24_) were reported as 48 mg·h^−1^·L^−1^ after 25 + 5 weeks of gestation, 106 mg·h^−1^·L^−1^ after 35 + 5 weeks of gestation and 203 mg·h^−1^·L^−1^ 18 weeks post-partum, with corresponding increases in C_max_ [24]. While the AUC_0–24_ in the second trimester of pregnancy was considerably lower than expected, exposures appeared to increase four-fold by the time of post-partum sampling, which may explain why linezolid was eventually discontinued five months post-partum due to polyneuropathy.

Minimum plasma concentrations (C_min_) as well as AUC influence linezolid-related adverse effects as well as clinical response to treatment [38]. Among 76 non-pregnant adult MDR/RR-TB patients in Georgia, after 4–6 weeks of 600 mg daily dosing of linezolid, median C_min_ was 0.235 mg/L (IQR 0.069–0.529) and median AUC_0–24_ was 89.6 mg·h/L (IQR 69–116.2) [39]. Jeyakumar et al. reported that, among 18 non-pregnant adults receiving 600 mg daily linezolid for MDR/RR-TB over 24 weeks in India, median AUC_0–24_ and C_min_ increased significantly between 6 and 18 weeks of dosing (184.2 to 233.2 mg·h/L and 1.98 to 3.16 mg/L, respectively), along with a progressive reduction in drug clearance during prolonged treatment with linezolid [40]. Linezolid is well known for its severe toxicity profile (Table 1), and changes in drug exposure impact not only the treatment efficacy but also the potential risk of serious and severe adverse events [41].

Although linezolid has been detected in breastmilk, pharmacokinetic data from three case reports suggest that systemic exposure in the infant is minimal [8]; the risk of sub-therapeutic doses in the context of neonatal *M. tuberculosis* infection is a potential but unvalidated concern.

## 4. WHO Group B Drugs

### 4.1. Clofazimine

Primarily used in leprosy treatment until its inclusion in the 9–11-month standardised regimen for MDR/RR-TB, this riminophenazine probably works through disrupting the production of mycobacterial ATP [42]. The drug is highly lipophilic and highly protein-bound, with a huge volume of distribution, which may or may not be significant in pregnancy as plasma protein concentrations are decreased while fat stores are increased [10]. The long terminal half-life (estimated to be significantly longer in women (49.5 days) than men (21.8 days)) leads to considerable delays in achieving steady-state concentrations at the currently recommended 100 mg daily dose in adults [43]. The optimal dosing of clofazimine in adults remains uncertain, and pharmacokinetic data among patients established on treatment for MDR/RR-TB remain limited.

Modelled data from South Africa show that AUC_0–24_ and C_max_ are higher after two months than after two weeks of dosing, and overall exposures appear to be much lower in women than in men—these findings suggest that an initial loading dose, with longer loading periods for patients with higher fat mass, may improve the efficacy of clofazimine without significantly increasing the risk of QT interval prolongation during treatment for MDR/RR-TB [42,43].

Reported pharmacokinetic properties of clofazimine in the treatment of tuberculosis have been well summarised in a recent systematic review by Stadler et al. [42], but the only reference to pregnancy was a small study of eight women with leprosy receiving variable doses of clofazimine (50–100 mg daily or 100 mg alternate days) while breastfeeding. The range of clofazimine plasma levels measured in breastmilk (0.8–1.7 mcg/mL) were higher than the mean (0.9 ± SD 0.03 mcg/mL) of the maternal plasma concentrations measured 4–6 h post dose [17]. However, despite this significant accumulation of clofazimine in breastmilk, the estimated dose ingested by the breastfed infant was <0.3 mg/kg/day, much less than the currently recommended minimum therapeutic dose of 2 mg/kg/day. Interestingly, a recent investigational modelling study has shown that, as human milk enhances the solubility of clofazimine, breastmilk may be used as a delivery vehicle for infants requiring therapeutic doses of the drug [44]. As breastfed infants appear to be exposed to much lower mg/kg doses than are associated with QT interval prolongation in adults receiving 100–300 mg clofazimine [45], the potential QT-prolonging effect in breastfed infants is unlikely to be a significant safety concern at current doses.

A few other case reports of clofazimine exposure among women successfully treated for leprosy or tuberculosis during pregnancy indicate that clofazimine crosses the placenta (as evidenced by infant skin discolouration), but no teratogenic effects have been documented [46,47,48]; neonatal pharmacokinetic parameters for clofazimine have not been reported [8].

### 4.2. Terizidone

Cycloserine and its pro-drug, terizidone, have long been used in the treatment of MDR/RR-TB and work through inhibiting mycobacterial cell wall synthesis [49]. There is usually a delay in absorption of this drug, up to six hours after dosing, resulting in a prolonged half-life [11]. Cycloserine is primarily excreted by the kidneys; therefore, drug exposure is affected by changes in renal function (with potentially increased drug clearance during pregnancy [10]).

While pharmacokinetic parameters have been described in several studies of healthy volunteers, few have reported on the pharmacokinetics among patients receiving multidrug treatment for MDR/RR-TB. In a cohort of 32 patients, aged ≥16 years, receiving mean daily cycloserine doses of 8.8 mg/kg (SD ± 3.3) (administered as 250 mg either once, twice or three times daily, depending on weight and creatinine clearance), serum concentrations after five days of exposure, measured at two and six hours post dose, were reported as 19.7 mcg/mL (range 7.1–43.4) and 18.1 mcg/mL (range 5.4–42.6) [50]. Serum concentrations in one fifth of these patients were higher after six hours than at two hours, reflecting the delayed absorption of the drug, but overall, concentrations were lower than the target peak serum concentrations of 20–35 mcg/mL [11]. There are no published pharmacokinetic data to suggest whether similar dosing in pregnancy would result in even lower exposures.

Recent population pharmacokinetic modeling has indicated that current recommended adult weight-based doses of cycloserine (500–750 mg daily) are adequate for an optimal bacterial kill rate at MICs < 16 mg/L [51], although this might not be sufficient for some *M. tuberculosis* strains as the probable epidemiological cutoff ranges between 32 and 64 mg/L [49]. An earlier model of cycloserine exposures among >200 adults with MDR/RR-TB suggested that daily doses of >1000 mg (exceeding current recommendations) may be required for optimal efficacy at MICs ≥ 16 mg/L and, as increased doses lead to increased plasma concentrations and increasing concern for drug-related neuropsychiatric adverse effects [11], the authors suggest that dividing the daily doses may mitigate these effects while maintaining adequate drug exposure [49].

In some countries, cycloserine is administered as terizidone, assuming similar efficacy, although it is unclear if dosing is equivalent as no therapeutic randomised trials have compared the two directly. Among 35 non-pregnant adults receiving terizidone for MDR/RR-TB, the measured cycloserine exposures after at least two weeks of terizidone (median dose 14.4 mg/kg (IQR 13.4–16.0), administered as 750 mg, 500 mg or 250 mg daily, based on weight and creatinine clearance) were reported as follows: a median C_max_ of 38.1 mcg/mL (IQR 32.6–47.2) and a median AUC over 10 h (AUC_0–10_) of 319 mcg·h^−1^·mL^−1^ (IQR 267.5–378.7) [52]. Terizidone is not specifically contraindicated in pregnancy or post-partum and, while there have been a few case reports and observational studies of patients receiving cycloserine/terizidone for the treatment of MDR/RR-TB while pregnant, no relevant pharmacokinetic data from maternal/infant plasma have been published.

A small case series of women and their infants exposed to cycloserine in utero and through breastfeeding indicate that the drug is largely well tolerated in pregnancy, and no teratogenic effects were identified [8,48]. In reference to a review on breastfeeding and exposure to antituberculosis drugs, Algharably et al. calculated from a mean breastmilk cycloserine concentration of 13.4 mcg/mL that the nursing infant would have been exposed to only 20% of the therapeutic dose [8]. This again raises theoretical concerns for subtherapeutic exposure in an infant infected with *M. tuberculosis;* however, the infant would be unlikely to experience significant side effects of the drug at such low doses.

## 5. Conclusions

As Group A and B drugs become more widely used in the treatment of MDR/RR-TB during pregnancy and post-partum, more pharmacokinetic data and development of appropriate population pharmacokinetic models, with limited sampling strategies, may assist in establishing the optimal dosing of these important antituberculosis medications in this often-neglected and vulnerable population. Furthermore, recommendations for safety monitoring and the management of specific adverse events among pregnant MDR/RR-TB patients and their infants exposed to Group A and B antituberculosis drugs may assist in improving maternal and infant tolerability and acceptability of MDR/RR-TB treatment during the ante- and post-partum periods.

## Figures and Tables

**Table 1:** World Health Organization grouping of antituberculosis drugs, with drug-specific safety profiles, monitoring and special considerations during pregnancy and post-partum.

Drug Name	Safety Profile *	Monitoring	Special Considerations during Pregnancy and/or Post-Partum
Group A drugs			
Levofloxacin (LFX)	Well tolerated. Diarrhoea, nausea, bloating, arthralgia. Mild QTc interval prolongation (less than for moxifloxacin), altered glycaemia, tendon rupture. Uncommon: peripheral neuropathy, change in mood or behaviour, insomnia, aortic dissection.	*Baseline*: ECG and electrolytes. *Through treatment*: Regular clinical assessment of symptoms (including cardiac symptoms: chest pain, dizziness, syncope, palpitations) with repeat ECGs if clinically indicated.	No reported changes in safety profile specific to pregnancy. No apparent increase in congenital malformations following in-utero exposure, despite historical concerns regarding foetal cartilage development [13].
Moxifloxacin (MFX)	Well tolerated. Diarrhoea, nausea, bloating, arthralgia. Significant QTc interval prolongation (10–20 ms), headaches, dizziness, altered glycaemia, tendon rupture. *Uncommon*: peripheral neuropathy, change in mood or behaviour, disturbance in mental abilities, aortic dissection.	*Baseline*: ECG and electrolytes. *Through treatment*: Monthly ECG with QTcF calculation, regular clinical assessment of symptoms (including cardiac symptoms: chest pain, dizziness, syncope, palpitations).	No reported changes in safety profile specific to pregnancy. Similar foetal concerns and findings as for levofloxacin above. ECG monitoring in breastfed infants may be warranted given the historical reports of older quinolone concentrations in breastmilk [14] but no infant plasma concentration data are available to support this concern for MFX.
Bedaquiline (BDQ)	Well tolerated. Nausea, arthralgia, headaches. QTc interval prolongation (10–15 ms, peak effect at week 15). *Uncommon*: hyperuricaemia, phospholipidosis, elevated transaminases (early signal of risk for pancreatitis).	*Baseline*: ECG, electrolytes and liver function tests. *Through treatment*: Monthly ECG with QTcF calculation, regular clinical assessment of symptoms (including cardiac and abdominal symptoms), with repeat liver function tests if clinically indicated.	No reported changes in safety profile specific to pregnancy. Evidence suggests that infants exposed to BDQ in-utero may have lower birthweight (but better health outcomes at 12 months) than those not exposed to BDQ during maternal MDR/RR-TB treatment [15]. BDQ accumulates in breastmilk [16]; consider ECG monitoring in infants while exposed to BDQ through breastfeeding (no published data on safety of BDQ in children under five years of age).
Linezolid (LZD)	Poorly tolerated. Nausea, vomiting, diarrhoea, myelosuppression, peripheral neuropathy. Optic neuritis, pseudomembranous colitis, vaginal candidiasis, hypoglycaemia, serotonin syndrome and lactic acidosis, tachycardia, transient ischaemic attacks, pancreatitis, seizures. *Uncommon*: Stevens-Johnson syndrome, angioedema, alopecia.	*Baseline*: Full blood count with differential white cell count, visual acuity and peripheral neuropathy screening. *Through treatment*: Frequent haemoglobin, platelet and neutrophil measurement in the first two months of exposure and then monthly or as clinically indicated; monthly assessment of vision and peripheral neuropathy symptoms.	Longer dosing duration resulting in increased plasma concentrations and drug exposure may potentially exacerbate the relatively common conditions of peripheral neuropathy and anaemia in pregnancy—closer monitoring is warranted. No evidence of teratogenic effects in humans. Low linezolid levels in breastmilk are unlikely to be harmful to nursing infants but concerns for adverse effects cannot be ruled out [8]. No routine monitoring is currently recommended, but assessment of full blood count and differential in the infant might be clinically indicated.
***Group B drugs***			
Clofazimine (CFZ)	Well tolerated. Hyperpigmentation/discolouration of skin, conjunctivae and bodily fluids; dry skin and itching. QTc interval prolongation (10–20 msec)—later effect due to long half-life. *Uncommon*: photosensitivity, abdominal pain with obstruction or bleeding (deposition of drug in intestinal mucosa).	*Baseline*: ECG and electrolytes. *Through treatment*: Monthly ECG with QTcF calculation, regular clinical assessment of symptoms (including cardiac and abdominal symptoms), regular counselling regarding likelihood of skin discolouration (may worsen over time, may be noticeable in newborn).	Theoretical risk that skin hyperpigmentation may be exacerbated by concentration of CFZ in increased body fat stores during pregnancy, however, no definitive data to support this. CFZ crosses the placenta and accumulates in breastmilk. Effect on infant from breastmilk exposure only is unclear. Skin discolouration has been documented in newborns following in-utero exposure [17]. QT interval prolongation is additive when CFZ is used with BDQ and/or MFX, therefore ECGs in breastfeeding infants and closer maternal ECG monitoring may be warranted in these cases.
Cycloserine (CS)/ Terizidone (TRD)	Variable tolerance, poorly tolerated by some patients. Poor concentration, lethargy, neuropathy, depression, psychosis. Seizures, jaundice, suicidal ideation, skin problems.	*Baseline*: NPAE screening. *Through treatment*: Monthly NPAE screening, regular clinical assessment of other symptoms.	No reported changes in safety profile specific to pregnancy. Potentially more susceptible to NPAEs post-partum when depressive symptoms may occur, but no published data. Increase NPAE monitoring may be warranted if co-administered with delamanid.
***Group C drugs***			
Ethambutol	Well tolerated	Not reviewed, but this drug is included in the standardised 9-month regimen recommended by WHO for use in pregnancy	
Delamanid	Well tolerated in adults	Not reviewed	
Pyrazinamide	Variable tolerance	Not reviewed, but this drug is included in the standardised 9-month regimen recommended by WHO for use in pregnancy	
Amikacin	Badly tolerated	Not reviewed, but this drug is NOT recommended for use in pregnancy due to unacceptable risk of ototoxicity and congenital deafness *	
Ethionamide/prothionamide	Poorly tolerated	Not reviewed, but this drug is NOT recommended for use in pregnancy due to increased risk of nausea, vomiting, hypothyroidism (maternal and foetal) and possible teratogenicity *	
Meropenem/imipenem-cilastatin	Poorly tolerated	Not reviewed	
*Para*-amino salicylic acid (PAS)	Poorly tolerated	Not reviewed	
***Uncategorised drugs***			
High-dose isoniazid	Well tolerated	Not reviewed, but this drug is included in the standardised 9-month regimen recommended by WHO for use in pregnancy	
Pretomanid	Well tolerated	Not reviewed	

* Taken from the Web Annexes of the World Health Organization operational handbook on tuberculosis (Module 4: Drug-resistant tuberculosis treatment), 2022 update [18]. ECG = electrocardiogram; msec = millisecond; NPAE = neuropsychiatric adverse events; TSH = thyroid stimulating hormone; QTcF = QT interval corrected for heart rate using Frideriecia’s cube root formula.

## Data Availability

This is not relevant for this manuscript as this was a literature review paper and not a study involving collection of data.
